# Specificity of the HIV-1 Protease on Substrates Representing the Cleavage Site in the Proximal Zinc-Finger of HIV-1 Nucleocapsid Protein

**DOI:** 10.3390/v13061092

**Published:** 2021-06-08

**Authors:** János András Mótyán, Márió Miczi, Stephen Oroszlan, József Tőzsér

**Affiliations:** 1 Laboratory of Retroviral Biochemistry, Department of Biochemistry and Molecular Biology, Faculty of Medicine, University of Debrecen, 4032 Debrecen, Hungary; motyan.janos@med.unideb.hu (J.A.M.); miczimario@med.unideb.hu (M.M.); 2 Doctoral School of Molecular Cell and Immune Biology, University of Debrecen, 4032 Debrecen, Hungary; 3 HIV Dynamics and Replication Program, National Cancer Institute, Frederick, MD 21702, USA; stephen.oroszlan@nih.gov

**Keywords:** human immunodeficiency virus, HIV-1, protease, viral proteases, specificity, viral proteins, nucleocapsid protein, retroviruses, substrate specificity

## Abstract

To explore the sequence context-dependent nature of the human immunodeficiency virus type 1 (HIV-1) protease’s specificity and to provide a rationale for viral mutagenesis to study the potential role of the nucleocapsid (NC) processing in HIV-1 replication, synthetic oligopeptide substrates representing the wild-type and modified versions of the proximal cleavage site of HIV-1 NC were assayed as substrates of the HIV-1 protease (PR). The S1′ substrate binding site of HIV-1 PR was studied by an in vitro assay using KIVKCF↓NCGK decapeptides having amino acid substitutions of N17 residue of the cleavage site of the first zinc-finger domain, and in silico calculations were also performed to investigate amino acid preferences of S1′ site. Second site substitutions have also been designed to produce “revertant” substrates and convert a non-hydrolysable sequence (having glycine in place of N17) to a substrate. The specificity constants obtained for peptides containing non-charged P1′ substitutions correlated well with the residue volume, while the correlation with the calculated interaction energies showed the importance of hydrophobicity: interaction energies with polar residues were related to substantially lower specificity constants. Cleavable “revertants” showed one residue shift of cleavage position due to an alternative productive binding mode, and surprisingly, a double cleavage of a substrate was also observed. The results revealed the importance of alternative binding possibilities of substrates into the HIV-1 PR. The introduction of the “revertant” mutations into infectious virus clones may provide further insights into the potential role of NC processing in the early phase of the viral life-cycle.

## 1. Introduction

The role of the protease (PR) of human retroviruses in the late phase of virus replication by processing the Gag and Gag-Pol polyproteins has been well established (for a review, see [1]). The viral polyprotein is translated from different open reading frames (ORFs), and the Gag polyprotein consists of the major structural proteins: the matrix (MA), the capsid (CA), the nucleocapsid (NC), and the p6 at the C terminus. The polyprotein containing the precursors of the viral enzymes, the reverse transcriptase (RT), the integrase (IN), and the protease is translated from the *pol* gene, while the ORF of *env* encodes the surface glycoproteins. The mature homodimeric PR is formed after its release from the precursor polyprotein by autoprocessing, and then the protease specifically cleaves the Gag and Gag-Pol polyproteins at well-defined sites. The rate of the limited proteolysis is not equivalent at the different sites, and this enables a sequential order of cleavages. The polyprotein cleavage is absolutely necessary for viral infectivity, which served as a rationale to design protease inhibitors as chemotherapeutic agents in order to block human immunodeficiency virus type 1 (HIV-1) infection and treat associated diseases, including acquired immunodeficiency syndrome (AIDS) [2].

The NC protein of HIV-1 is a small basic protein containing two zinc-fingers. The NC has a variety of functions in viral replication as it is involved in the cDNA synthesis, dimerization, maturation, and packaging of genomic RNA, virus assembly, and possesses nucleic acid chaperone activity [3].

**Figure 1:**
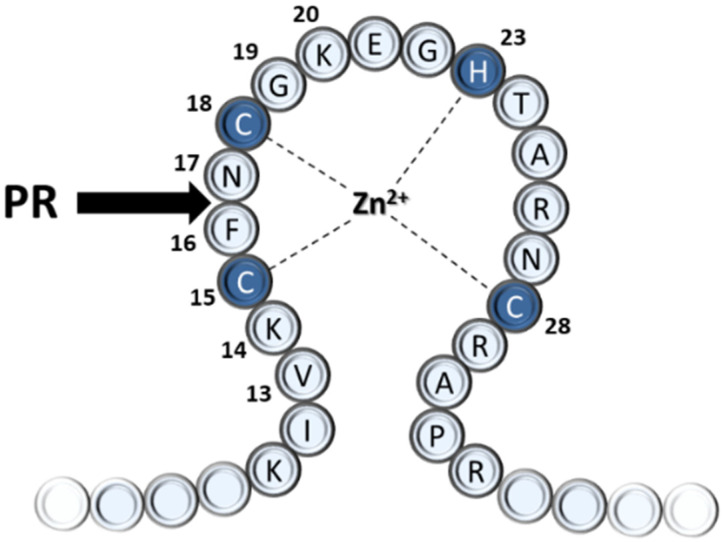
The N-terminal sequence of the first (proximal) zinc-finger of the HIV-1 nucleocapsid protein. The sequence is numbered, the Cys and His residues involved in zinc binding are coloured by dark blue. The black arrow indicates the natural cleavage site of PR.

Based on in vitro experiments with purified cores of equine infectious anemia virus (EIAV), a role of the PR in the early phase was proposed by cleaving the NC protein at the zinc-fingers [4]; the biochemistry of these cleavages has been published [5]. Later studies demonstrated that the HIV-1 PR is also part of the viral core entering the target cell [6]. Oligopeptides representing the predicted cleavage sites in the first zinc-fingers (NC-1) of human immunodeficiency virus (HIV) type 1 strain IIIB (HIV-1_IIIB_) and HIV-2_ROD_ NC proteins were substrates of the PRs. Based on the sequence homology with EIAV, originally, the oligopeptide substrate was predicted to be cleaved between Cys and Phe residues of the sequences representing the HIV-1 NC-1 cleavage site (KIVKCFNCGK) [6], but later, it was proved that the cleavage occurs one residue further from the expected place [7], between Phe and Asn residues (F16 and N17) of the first zinc-finger domain (Figure 1). Studies with chemically synthesized or recombinant proteins later also confirmed the shifted cleavage site [8,9].

Peptides representing the predicted cleavage sites in the second zinc-fingers were not substrates of the HIV-1 PR [8]; however, in vitro studies indicated another site of cleavage in the second zinc-finger [9]. Even though the cleavage within the retroviral NC zinc-fingers occurs in vitro in the presence of EDTA, labelled antibodies of the amino- and carboxyl-terminus of NC appeared to bind at different localizations in the nucleus of murine leukaemia virus-infected cells [10].

Based on the above-mentioned findings, the potential role of the PR in the early phase of the retroviral life-cycle was suggested, either by performing post-maturation cleavages of NC and CA or by cleaving protein substrates [1]. As compared to the function in the late phase events, the role of the PR in the early phase is less well established and is still controversial. Besides the inhibition studies that revealed effects of PR inhibition on early phase events [11,12,13,14,15], results that do not support the essential activity of HIV-1 PR in the early steps of replication have also been reported [16,17,18].

Similar to the other retroviral NC proteins, the zinc-finger sequence motifs of HIV-1 NC are also highly conserved [19]; one of the possible reasons for the high conservation was proposed to be the processing of NC at this cleavage site [9]. The naturally occurring virus variants contain no mutations at the cleavage position (F16 and N17 residues), but multiple studies investigated the effects of mutations at these sites (e.g., N17 mutants). For example, the genetically engineered N17K mutant virions were found to show increased transduction ability, in agreement with the enhanced RNA packaging as compared to the wild-type [20]. In contrast to this, another study revealed an ~10,000-fold decrease in infectivity for N17K mutant as compared to the wild-type, while the mutation did not block viral replication and stimulated RNS packaging [21], but a neutral nature of N17K mutation was also published [22]. Thomas et al. found that none of the N17A, N17G, N17F, N17L, N17K, N17R, and N17S mutations had a major effect on reverse transcriptase activity and did not impair the production and release of virion particles, but N17F and N17G mutants were replication-defective [23]. The virus bearing the N17A mutation was found to retain its ability for replication, similar to the wild-type [24]. Additionally, this mutant was found previously to be sensitive for proteolysis [9]. Nevertheless, the role of the proteolytic processing of NC at the proximal zinc-finger in the early steps of replication has not been elucidated unequivocally to date, even though its importance in the viral life-cycle has been implied by the high conservation of the cleavage site and by those studies, which revealed interference of NC mutations with replication and infectivity.

In order to study the proteolytic processing of HIV-1 NC protein and its role in the early phase of replication, recombinant HIV viruses are required containing mutations at the cleavage site of the NC. To facilitate the design of such mutant viruses, we studied the effect of various mutations in the P1′ position of the NC protein cleavage site yielding peptides more susceptible as well as more resistant to proteolysis by HIV-1 PR. The set of the previously studied P1′ mutants—used in in vitro processing and viral studies [9,23,25]—was not sufficient enough to obtain detailed information about the PR specificity; therefore, here we extended those preliminary in vitro studies and also investigated the amino acid preferences in silico by determining the possible correlation between the size of the P1′ residue as well as the calculated substrate interaction energies and the determined activation energies. We have also studied the effect of second-site mutations on the cleavability of the peptides representing the zinc-finger cleavage site.

## 2. Materials and Methods

### 2.1. Oligopeptide Synthesis and Characterization

Oligopeptides were synthesized by solid-phase peptide synthesis on a Model 430A automated peptide synthesizer (Applied Biosystems, Inc., Foster City, CA, USA) using t-butoxycarbonyl (BOC) or 9-fluorenylmethyloxy-carbonyl chemistry and were purified by reversed-phase HPLC. The amino acid composition of the peptides was determined with a Beckman 6300 amino acid analyzer. Stock solutions and dilutions were made in 5 mM dithiothreitol (DTT), and the peptide concentrations were determined by amino acid analysis.

### 2.2. Enzyme Assay with Synthetic Peptide Substrates

The assay was performed in 0.25 M potassium phosphate buffer, pH 5.6, containing 7.5% glycerol, 1 mM EDTA, 5 mM DTT, in the presence of 2 M NaCl. The reaction mixture was incubated at 37 °C for 1 h and then stopped by the addition of guanidine-HCl (6 M final concentration). The solution was acidified with trifluoroacetic acid (TFA) followed by an injection of an aliquot onto a Nova-Pak C_18_ RP-HPLC column (3.9 × 150 mm). The substrates and the cleavage products were separated using an increasing water–acetonitrile gradient (0%–100%) in the presence of 0.05% TFA. The composition of the cleavage products was determined by amino acid analysis. Kinetic parameters were determined at less than 20% substrate turnover by fitting the data to the Michaelis–Menten equation by using the Fig. P program (Fig. P Software Corp., Durham, NC, USA). The range of substrate concentration was 0.02–2.0 mM, depending on the approximate K_m_ values. The standard errors of the kinetic parameters were below 20%. Active-site titration of HIV-1 PR was performed with BOC-Val-Val-Phe-Phe-Val-Val-NH_2_, a phosphinate transition state analogue inhibitor that was synthesised by Grobelny et al. [26].

### 2.3. Molecular Modeling

A high-resolution crystal structure of HIV-1 PR was used (Protein Data Bank accession code: 1K1T, [27]) for modelling of the enzyme-substrate complexes with peptides in Table 2. Models for KIVKCF↓NCGK substrate (peptide 1 in Table 2) and its P1′ substituted versions (peptide 2–14 in Table 2) were built into the active site of HIV-1 PR by replacing the inhibitor. The complexes were minimized on Silicon Graphics O2 or Fuel workstations using the Sybyl software package (Tripos Inc., St. Loius, MO, USA).

Initially, only the substrate was allowed to move with distance constraints from backbone nitrogen and oxygen atoms to the appropriate atoms of the fixed enzyme structure, simulating the conserved hydrogen bond network between the enzyme and the substrate/inhibitor. After 100 Simplex and 100 Powell iterations, the enzyme structure was relaxed, and the force constants of hydrogen bond constraints were gradually decreased from 200 to 0 kcal·mol^−1^·angstrom^−2^ during the following 200 Powell iterations. All constraints, except distance constraints between oxygen atoms of side-chains of the 2 catalytic aspartates, were removed, and another 500 iterations were applied. Kollman all-atom force field [28] implemented in Sybyl was used, the dielectric constant was set to 4, while other parameters were the default values of Sybyl.

At the end of minimization, ΔE_Xaa_ interaction energies were calculated between enzyme and substrate and compared to the corresponding value of the P1′ Gly substituted peptide. The calculated ΔΔE_calc_ = ΔE_Gly_ − ΔE_Xaa_ values were correlated with the experimentally determined ΔΔG_exp_ = −RT ln ((k_cat_/K_m_)_Xaa_/(k_cat_/K_m_)_Gly_) relative activation energies. The ΔΔE_calc_ values were corrected with the ΔΔE_solv_ relative solvation energies [29]. ΔΔE_solv_ = ΔE_Solv(Xaa)_ − ΔE_Solv(Gly)_, where ΔE_Solv(Xaa)_ = 2.303RTπ derived from the experimentally determine π value of hydrophobicity [30]. The ΔΔG_exp_ values were also plotted against the measured volumes of the amino acid residues [31].

### 2.4. Design of Recombinant Fluorescent Protein Substrates

The cloning cassette of the primarily designed pDest-His_6_-MBP-mApple plasmid [32] was modified in this work to contain a (GGGGS)_4_ flexible linker prior to the fluorescent tag, using the method for the introduction of a new cloning cassette described previously [33]. The empty pDest-His_6_-MBP-(GGGGS)_4_-mApple plasmid was then linearized with BamHI and PacI restriction endonucleases (New England Biolabs), and the oligonucleotide primers coding for cleavage sites of HIV-1 PR (Table 1) were cloned into the expression vector using the previously described protocols [33]. The success of cloning was verified by sequencing. The inserted sequences represented the wild-type and modified forms of the entire proximal zinc-finger (11-KIVKCFNCGKEGHTARNCRAPR-32).

### 2.5. Expression, Purification and Cleavage of Protein Substrates

The recombinant fluorescent substrates were expressed in *E. coli* BL21(DE3) cells and purified using Ni-NTA magnetic agarose beads as it was described previously [32,33,34,35]. The purified substrates were concentrated by 10 K Amicon Ultra 0.5 mL centrifugal filters (Merck Millipore, Burlington, MA, USA) while changing the buffer to distilled water.

The purified fluorescent proteins were used as substrates for HIV-1 PR. The reaction mixtures contained 16 µL buffer (150 mM sodium chloride, 100 mM sodium acetate pH 5.5, containing either 1.6 mM ZnCl_2_ or 10 mM DTT and EDTA), 2 µL recombinant protein substrate (0.1 mg/mL final concentration), and 2 µL HIV-1 PR (250 nM final concentration). The cleavage reactions were incubated for 16 h at 37 °C and were stopped by t he addition of sample buffer (300 mM Tris, 20% glycerol, 0.1% bromophenol blue) lacking SDS and β-mercaptoethanol. The uncleaved substrates and cleavage products were separated by polyacrylamide gel electrophoresis (PAGE) using 14% gel. After native PAGE, the gel was washed with distilled water for 30 min, and the proteins were detected by blue-light transillumination using Cleaver safeVIEW (Cleaver Scientific Ltd, Warwickshire, UK) gel documentation system. The GelAnalyzer program—developed by István Lázár at the Department of Inorganic and Analytical Chemistry at University of Debrecen—was used for densitometry (www.gelanalyzer.com, accessed on 1 April 2021).

## 3. Results

### 3.1. Probing the S1′ Binding Site of HIV-1 Protease Using Substituted Peptides Representing the Proximal Cleavage Site of HIV-1 NC

To probe the amino acid preferences of the S1′ binding site of HIV-1 protease, we have introduced various P1′ mutations into KIVKCF↓NCGK decapeptide substrate representing the sequence of proximal zinc-finger cleavage site (NC-1) (Table 2). The kinetic constants for the cleavage of the substituted peptides by HIV-1 PR were determined (Table 2).

As was expected, we obtained a higher k_cat_/K_m_ value for N17L, N17V, and N17A mutants having small or medium-sized hydrophobic residue in the P1′ position. The N17D mutation prevented substrate processing, while all other mutants retained their susceptibility for proteolytic processing, and the lowest k_cat_/K_m_ value was obtained for the N17G mutant. The peptides having a long flexible and polar side-chain (N17R and N17K mutants) the catalytic constants were found to be comparable or lower as compared to the wild-type, respectively; both mutations increased the K_m_ (Table 2). The N17F mutation was found to decrease K_m_ and increase k_cat_, and the specificity constant (k_cat_/K_m_) determined for this mutant was remarkably higher than for the wild-type.

**Figure 2:**
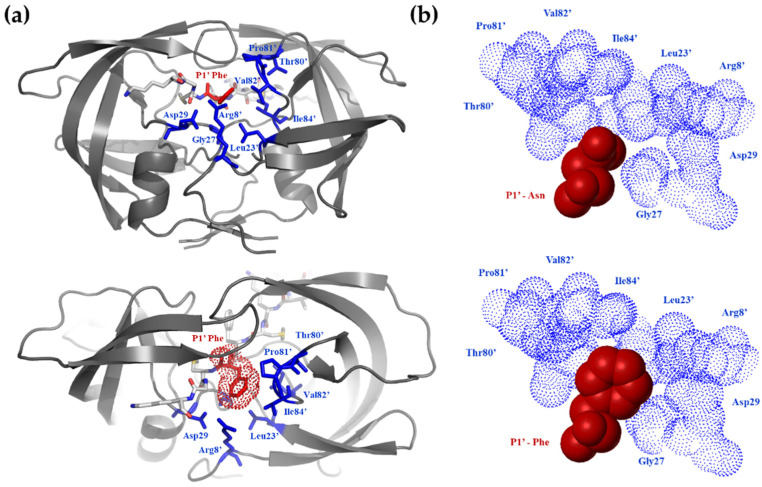
Fitting of Asn and Phe residues into the S1 substrate binding site of HIV-1 protease. (**a**) The structure of HIV-1 PR complexed with KIVKCFFGCK oligopeptide substrate. The S1′ and P1′ residues are shown by blue and red colours, respectively. (**b**) In contrast to the wild-type P1′-Asn, the P1′-Phe residue of mutant NC-1 sequence was expected to provide favourable hydrophobic interactions at the S1′ subsite.

A wide range of kinetic constants was observed, suggesting that the enzyme is very sensitive to the substrate side-chain being at the P1′ position. Although P1′ specificity of HIV-1 PR was studied previously, probing other substrate sequences [36,37], the results of those studies are different, as expected from the highly context-dependent nature of the HIV-1 PR specificity [38,39,40]. In other terms, the P1′ specificity is a function of the residues surrounding this position. Based on previous studies, the S1′ site of HIV-1 PR is large and hydrophobic [40]; therefore, the P1′-Asn is not expected to provide optimal hydrophobic interactions (Figure 2).

**Figure 3:**
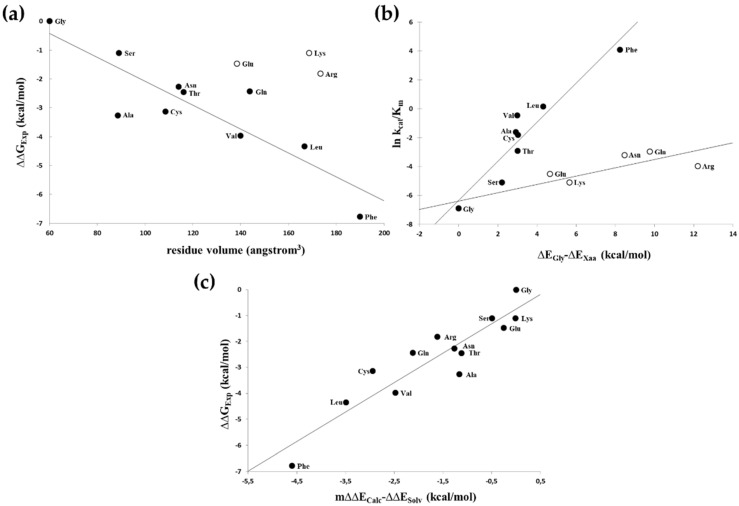
Plotting experimentally determined relative activation energies and calculated S1′-P1′ relative interaction energies. (**a**) Volumes of non-charged P1′ residues (solid circles) correlated well (r^2^ = 0.76) with the experimentally determined relative activation energies, while charged P1′ residues (open circles) were kinetically substantially less favourable than non-charged residues having similar sizes and were excluded from the correlation. (**b**) Interaction energies calculated for nonpolar (solid circles) and polar/charged (open circles) residues gave separate correlations with the specificity constants (r^2^ = 0.88 and r^2^ = 0.73, respectively). (**c**) Correlation of calculated S1′-P1′ relative interaction energies corrected for the solvation energy with the experimentally determined relative activation energies. Calculated relative interaction energies were multiplied by an empirical value (m = 0.25) and corrected with the relative solvation energies as described in the text gave a good correlation (r^2^ = 0.85) with the relative activation energies.

The Phe side-chain in the P1′ position provided the best van-der Waals contacts (Figure 2b) and, by far, was the most efficient substrate (Table 2). A good correlation (r^2^ = 0.76) was observed between the relative activation energies derived from the catalytic constants (relative to that of the P1′-Gly containing peptide) and the size of the P1′ side-chain of uncharged residues (Figure 3a). When the calculated substrate-binding energies relative to that of the P1′-Gly containing peptides were correlated with the determined specificity constants, there was a segregation of the hydrophobic as well as non-hydrophobic residues with r^2^ = 0.88 and 0.73 values (Figure 3b). The substantially smaller slope obtained for the non-hydrophobic residues suggested that only a smaller portion of the interaction energy could be converted to activation energy in these cases, indicating a high preference for hydrophobicity at this position. When the calculated energy differences were corrected with the solvation energy [29] derived from the π value of hydrophobicity [30], a good correlation (r^2^ = 0.85) was obtained for all residues (Figure 3c).

### 3.2. Design of Nucleocapsid Cleavage Site “Revertants”

To demonstrate the importance of the sequence context on the site of cleavage and its efficiency, we have attempted to generate cleavage site “revertants,” in which the internal positions of the P1′ modified proximal zinc-finger motif (-Cys-Phe↓Gly-Cys-) are not changed. Nevertheless, cleavability within this region is regained by introducing mutations outside of the P2-P2′ region. Peptides having mutations that were predicted to regenerate proteolytic susceptibility are listed in Table 3. While a true “revertant” would be the G17N mutant, the variants studied here are also referred to as “revertants” but need to be considered as “pseudo-revertants.” All multiply substituted “revertant” cleavage site peptides used in this study contained the N17G mutation (peptide 15–20 in Table 3). Those peptides that were not cleaved at the selected threshold value (estimated k_cat_ < 0.01 s^−1^) were considered to be similar in cleavage susceptibility to the N17G mutant (peptide 14 in Table 3).

The primary target positions for the design of such “revertants” should be the residues adjacent to the -Cys-Phe↓Gly-Cys- motif since proper side-chains in these positions might substitute for the loss of interaction energy caused by the Asn→Gly mutation of P1′ residue. Based on previous detailed specificity studies on the HIV-1 PR, we have selected β-branched substituting residues of which side-chain can cause substrate side-chain rearrangements to provide a productive enzyme-ligand interaction (sequence context-dependence of the HIV-1 PR specificity was reviewed previously by Tőzsér and Oroszlan [1]).

K14T substitution, providing the corresponding residue of EIAV NC-1 [7], did not yield a cleavable peptide (peptide 15 in Table 3). However, substitution with the also β-branched but completely hydrophobic Ile provided a cleavable sequence, but the site of cleavage was shifted into the position analogous to the EIAV NC-1 cleavage sites (peptide 16 in Table 3). This peptide was about 10-fold better substrate than the wild-type one (peptide 1 in Table 3), and its specificity constant was comparable to those obtained for peptides representing the slowest maturation cleavage sites in Gag [9]. Previous studies indicated that the presence of Lys in position 14 in the wild-type HIV-1 NC-1 sequence (peptide 1 in Table 3) might be responsible for the lack of cleavage between C15 and F16 (positions corresponding to the previously established EIAV NC-1 cleavage site) since in such way of binding the K14-S2 interactions are prohibitive [8] as Lys at the P2 position of other substrate sequences prevented substrate hydrolysis [37,41].

**Figure 4:**
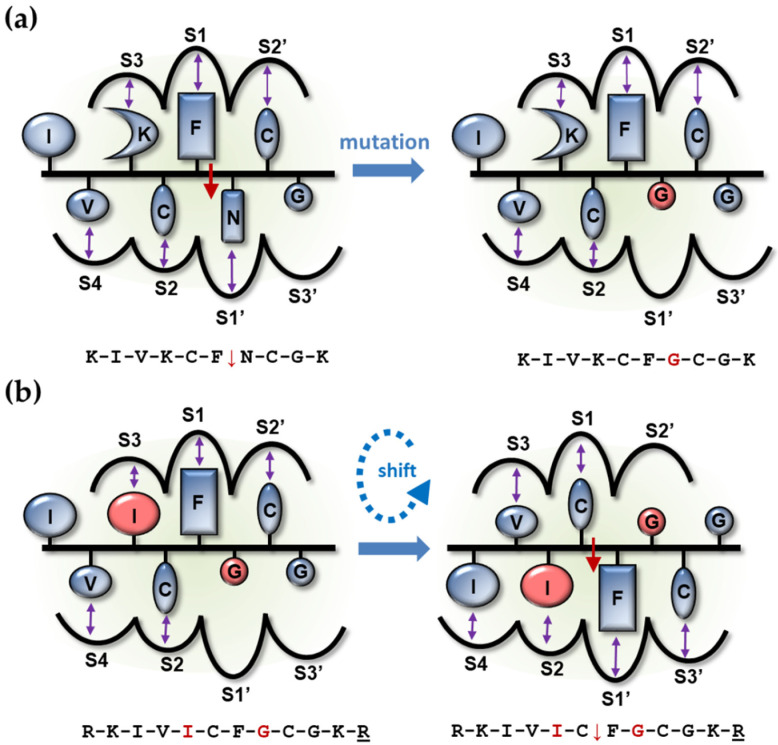
Predicted subsite interactions that lead to the shifted cleavage of the Ile for Lys-substituted peptide. Modified residues are highlighted in red. Favourable subsite interactions are indicated by purple arrows. The red arrow shows within the sequence if cleavage occurs. (**a**) The figure represents the productive binding mode of the peptide representing the naturally occurring proximal zinc-finger cleavage site in HIV-1 (peptide 1 in Table 3), leading to hydrolysis at the Phe↓Asn site. Upon P1′Asn→Gly mutation, the peptide is not cleaved (peptide 14 in Table 3). (**b**) The peptide substrate is not processed by cleavage between Phe and Gly residues of the double-substituted peptide (peptide 16 in Table 3) when Ile occupies the S3 binding subsite. For this peptide, the same Ile residue occupies the S2 binding subsite in the productive binding where cleavage occurs between Cys and Phe residues.

The shift of cleavage site due to an alternative productive binding caused by the P2-Ile substitution is demonstrated in Figure 4. The figure shows the productive binding of the substrate representing the wild-type sequence (peptide 1 in Table 3) in which Lys-S3, Cys-S2, Phe-S1, and Asn-S1′ interactions contribute to the binding energy. The Asn→Gly substitution in the P1′ position (peptide 14 in Table 3) removes the favourable Asn-S1′ interaction and prohibits efficient cleavage. The Lys→Ile substitution (peptide 16 in Table 3) does not compensate for this loss when the substrate binds to the enzyme in the original position (Figure 4b); however, it provides an alternative productive binding mode in which the peptide is rotated and therefore shifted one residue in the binding cleft of the PR, resulting in favourable Val-S3, Ile-S2, Cys-S1, and Phe-S1′ interactions. Based on inspection of the binding sites, such shift cannot be achieved by actual rotation of the ligand within the binding site (with an open flap of the enzyme); rather, it can only occur by a separate, shifted binding event of the ligand.

It is of interest to note that not only K14I mutation but also an Ile insertion following K14 resulted in a cleavable sequence with similar kinetic parameters (peptide 17 in Table 3), but substitution of the G19 to Ile did not provide a cleavable peptide (peptide 18 in Table 3). The favourable interactions provided in P3′-S3′ by this mutation for the -Cys-Phe↓Gly-Cys- motif cleavage apparently cannot compensate for the loss of S1′-P1′ interactions exerted by the Gly substitution of N17. The K14I/G19I double substitutions provided a substrate with a site of cleavage and specificity constant (peptide 19 in Table 3) identical to those obtained with the K14I mutant (peptide 16 in Table 3). Considering a Lys-Cys↓Phe-Gly cleavage, I19 would provide only P4′-S4′ interaction, which is—based on previous specificity studies—usually negligible, and therefore cannot compensate for the very unfavourable K14-S2 interactions. Interestingly, K14L mutation (peptide 20 in Table 3) also provided a cleavable sequence; however, analysis of the composition of the multiple cleavage products suggested the simultaneous cleavage at -Val-Leu↓Cys-Phe- and -Leu-Cys↓Phe-Gly-, indicating that two types of substrate binding could be productive in this case.

### 3.3. Cleavage Reactions Using Recombinant Protein Substrates

The processing of the proximal zinc-finger motif was studied using recombinant protein substrates, as well. We have studied previously the cleavage of the wild-type as well as the N17F and N17G mutant native NC proteins in a precursor-like recombinant protein (r-pre-NC) containing five cleavage sites [9]. Here, we describe His_6_-MBP-(GGGGS)_4_-mApple artificial substrates that contain only the sequence of the proximal HIV-1 NC zinc-finger. Besides the wild-type sequence (11-KIVKCFNCGKEGHTARNCRAPR-32), the N17T, N17L, N17F (Table 2), I14_ins-N17G and K14I-N17G (Table 3) mutants were also studied. These mutants were selected for measurements as they showed comparable (N17T) or higher (N17F, N17L, K14I-N17G, and I14_ins-N17G) k_cat_/K_m_ values as compared to that of the wild-type (Table 2).

To study the effect of zinc on the processing of recombinant fluorescent proteins, the cleavage reactions were performed in the presence of DTT and EDTA, and ZnCl_2_-containing buffer was also used. The EDTA was used as a chelator to provide an ion-free state of zinc-fingers (opened conformation) and DTT was added to reduce Cys residues and prevent the formation of intra- and intermonomeric disulphide bonds, while the zinc-containing buffer was used to enable the formation of zinc-fingers.

**Figure 5:**
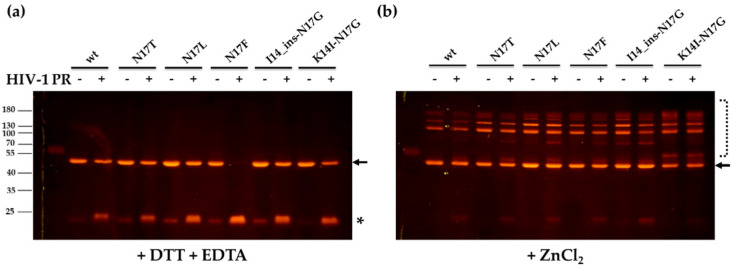
The cleavage of recombinant protein substrates with HIV-1 PR. Cleavage reactions were performed with HIV-1 PR, using buffers supplemented either with DTT and EDTA (**a**) or ZnCl_2_ (**b**). After the cleavage of recombinant substrates, the separation of substrates and cleavage products with PAGE was performed using non-denaturing conditions. The proteins were detected in the gel using blue-light transillumination. Arrows indicate bands of the uncleaved substrates, while asterisk indicates bands of the cleavage products containing the C-terminal mApple fluorescent protein. The dashed line (**b**) indicates putative oligomers.

The substrates were found to be cleaved by HIV-1 PR in the buffer containing DTT and EDTA (Figure 5a). The highest substrate turnover was observed for the N17F mutant, as it was expected based on the remarkably higher catalytic constant determined for this mutant as compared to the wild-type (Table 2). The results indicated that the cleavage sites are accessible in the ion-free zinc-fingers, and the chelation of zinc ions by EDTA and the reduction of Cys residues by DTT provides an opened conformation of the zinc-fingers. In contrast to this, the substrates were resistant to proteolysis in the presence of zinc, which indicated that the zinc-induced closed conformation made the proximal NC cleavage site inaccessible for the protease, preventing proteolysis (Figure 5b). We observed no processing for N17F mutant substrate in the zinc-containing buffer, and even it was completely digested in the DTT and EDTA-containing buffer. Our results are in agreement with the findings of Wondrak et al., who reported previously that the NC protein containing the wild-type NC-1 cleavage site is not processed in the presence of zinc [9]. Based on our results, none of the studied mutants prevented the formation of the zinc-finger structural motif and made the cleavage site accessible for the protease.

In the presence of ZnCl_2_, none of the substrates was processed by the PR, but fluorescent bands appeared both in the cleavage reactions and substrate control samples that were not observed in the samples containing DTT and EDTA (Figure 5b). We assume that these bands correspond to oligomers formed via intermolecular disulphide bonds, while the formation of such disulphide bridges was inhibited in the DTT and EDTA-containing buffer due to the reduction of Cys residues (Figure 5a).

**Figure 6:**
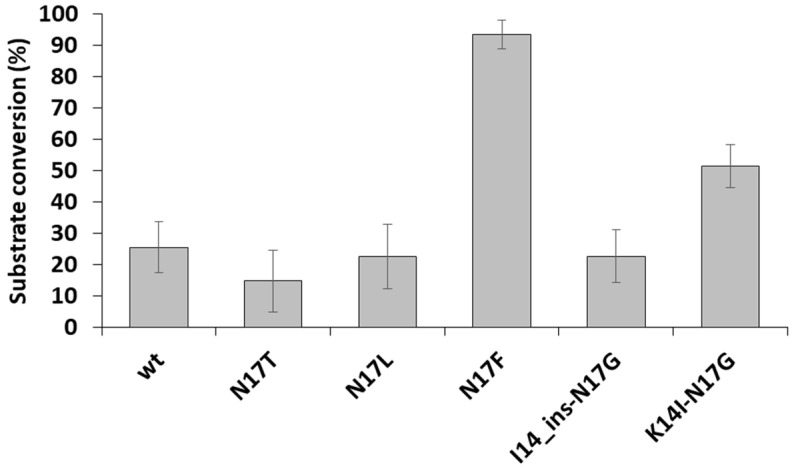
A comparison of relative cleavage efficiencies of recombinant substrates. The substrate conversion was determined based on the band intensities of uncleaved substrates and fluorescent cleavage products. *n* = 2.

After electrophoresis, the band intensities were determined by densitometry for the reactions performed in the presence of DTT and EDTA. We obtained the highest substrate conversion for the N17F mutant (Figure 6), in agreement with the highest catalytic constant determined for this mutant (Table 3). The substrate conversions obtained for the other mutants were more comparable, possibly due to the relatively lower sensitivity of the gel-based analysis. Furthermore, it should be noted that the fluorescent protein-based assays and the catalytic constant determinations were performed by using different substrates and reaction conditions. The determination of kinetic parameters by the fluorescent protein-based assay was not possible because the optimal conditions of cleavage reaction are not compatible with the Ni-NTA-based protease assay as DTT and EDTA impair substrate immobilization.

## 4. Discussion

In this work, the S1′ binding site specificity of HIV-1 protease was studied using a synthetic decapeptide substrate series representing the wild-type or P1′ modified sequence of the proximal zinc-finger cleavage site in HIV-1 (Table 2). P1′-Phe mutant was found to be the most efficient substrate for HIV-1 PR, as was expected. This is in agreement with the high preference of retroviral proteases for hydrophobicity at this position, due to the large cavity volume and hydrophobic nature of binding sites harbouring the cleavage site [39,40]. Preference for large and hydrophobic residues in the P1′ position was proved by molecular modelling calculations, as well (Figure 3). However, specificity studies revealed the very strong sequence context-dependence of the specificity of HIV-1 PR [1,40], the good correlation observed in this study between the relative activation energies and the volumes of uncharged P1′ residues (Figure 3) indicates that at least in this substrate context the perturbing effects of the neighbouring substrate residues are negligible.

“Revertant” oligopeptide substrates representing the sequence of the proximal zinc-finger cleavage site in HIV-1 were designed to study the NC cleavage. Designed mutations were predicted to regain the cleavability of the N17G mutant substrate (Table 3). A shift was observed in the position of cleavage compared to the naturally occurring Phe↓Asn cleavage site, and the Cys↓Phe peptide bond was cleaved in the case of all cleavable “revertants.” In this binding mode, the Phe residue binds to the S1′ site, while the Cys sidechain binds to the S1 in the case of all cleavable substrates summarized in Table 3. In the case of HIV-1 PR, seven more or less distinct substrate-binding subsites are involved in substrate recognition [42]. Therefore, a decapeptide substrate can bind to the enzyme in four different ways (assuming only one-directional binding possibility). Interestingly, we observed not only one but also two productive binding modes for K14L “revertant” substrate. Nevertheless, studying more than 200 decapeptide substrates, for HIV-1 we have not encountered such a double cleavage; therefore, it could be only a very rare event.

The original purpose of generating “revertants” was to provide additional possibilities to study the role of NC processing in the early phase of viral replication. It should be noted that the results of Thomas et al. [23] utilizing the limited set of cleavage site mutations [9] suggested that these mutations rather impact the late phase of the viral replication, despite the presence of the PR in the core of the virion [8], which implied its function in the early phases of the replication cycle. It is important to note that the studies on the early steps of replication may be limited by the small fractions of infectious virions entering the cells and by the difficulties of studying these events using the currently available methods and detection systems [1]. Additionally, it needs to be considered that the effects of NC mutations and PR inactivation by inhibitors may be cell type-specific and also dependent on the applied concentrations of PR inhibitors and infectious virions [23,43]. The correlation between the cleavage rates of oligopeptide substrates and virus infectivity may also be only apparent [25]; therefore, the replication efficiencies of “revertant” NC mutant-containing virions may be estimated with relatively low reliability purely based on the results of in vitro processing assays. The introduction of the “revertant” mutations into infectious virus clones may provide further insights into the potential role of NC processing in the early phase of the viral life-cycle. Nevertheless, the current study may still contribute to a better understanding of the retroviral PR specificity.

## Figures and Tables

**Table 1:** The sequences of oligonucleotide primers used for cloning and sequencing. Amino acid sequences of HIV-1 NC-1 cleavage sites in His_6_-MBP-(GGGGS)_4_-mApple substrates are indicated above the sequences of forward (FWD) and reverse (REV) oligonucleotide primer pairs. Modified residues are highlighted with grey background in the cleavage site sequences.

Primer Name	Cleavage Site Sequence/Oligonucleotide Primer Sequence
**Wild-type**	**KIVKCFNCGKEGHTARNCRAPR**
FWD	5′-TAAAAAAATTGTGAAATGCTTTAACTGCGGCAAAGAAGGCCATACCGCGCGTAACTGCCGTGCGCCGCGTG-3′
REV	5′-GATCCACGCGGCGCACGGCAGTTACGCGCGGTATGGCCTTCTTTGCCGCAGTTAAAGCATTTCACAATTTTTTTAAT-3′
**N17F**	**KIVKCFFCGKEGHTARNCRAPR**
FWD	5′-TAAAAAAATTGTGAAATGCTTTTTTTGCGGCAAAGAAGGCCATACCGCGCGTAACTGCCGTGCGCCGCGTG-3′
REV	5′-GATCCACGCGGCGCACGGCAGTTACGCGCGGTATGGCCTTCTTTGCCGCAAAAAAAGCATTTCACAATTTTTTTAAT-3′
**N17L**	**KIVKCFLCGKEGHTARNCRAPR**
FWD	5′-TAAAAAAATTGTGAAATGCTTTCTGTGCGGCAAAGAAGGCCATACCGCGCGTAACTGCCGTGCGCCGCGTG-3′
REV	5′-GATCCACGCGGCGCACGGCAGTTACGCGCGGTATGGCCTTCTTTGCCGCACAGAAAGCATTTCACAATTTTTTTAAT-3′
**N17T**	**KIVKCFTCGKEGHTARNCRAPR**
FWD	5′-TAAAAAAATTGTGAAATGCTTTACCTGCGGCAAAGAAGGCCATACCGCGCGTAACTGCCGTGCGCCGCGTG-3′
REV	5′-GATCCACGCGGCGCACGGCAGTTACGCGCGGTATGGCCTTCTTTGCCGCAGGTAAAGCATTTCACAATTTTTTTAAT-3′
**K14I-N17G**	**KIVICFGCGKEGHTARNCRAPR**
FWD	5′-TAAAAAAATTGTGATTTGCTTTGGCTGCGGCAAAGAAGGCCATACCGCGCGTAACTGCCGTGCGCCGCGTG-3′
REV	5′-GATCCACGCGGCGCACGGCAGTTACGCGCGGTATGGCCTTCTTTGCCGCAGCCAAAGCAAATCACAATTTTTTTAAT-3′
**I14_ins-N17G**	**KIVKICFGCGKEGHTARNCRAPR**
FWD	5′-TAAAAAAATTGTGAAAATTTGCTTTGGCTGCGGCAAAGAAGGCCATACCGCGCGTAACTGCCGTGCGCCGCGTG-3′
REV	5′-GATCCACGCGGCGCACGGCAGTTACGCGCGGTATGGCCTTCTTTGCCGCAGCCAAAGCAAATTTTCACAATTTTTTTAAT-3′
sequencing primer	5′-GATGAAGCCCTGAAAGACGCGCAG-3′

**Table 2:** Proteolytic processing of substituted oligopeptides representing the first cleavage site in the HIV-1 NC. The table shows the wild-type and P1′ single mutant oligopeptides. Modified positions are highlighted with a grey background. The fold-change of k_cat_/K_m_ values is also indicated.

	Name	Sequence	Cleavage Site	K_m_ (mM)	k_cat_ (s^−1^)	k_cat_/K_m_ (mM^−1^ s^−1^)	k_cat_/K_m_ Fold-Change
1.	wild-type	KIVKCFNCGK	CF↓NC	0.43 ^1^	0.017 ^1^	0.040 ^1^	-
2.	N17F	KIVKCFFCGK	CF↓FC	0.02 ^1^	0.900 ^1^	59.6 ^1^	1490.00
3.	N17L	KIVKCFLCGK	CF↓LC	0.17 ^1^	0.196 ^1^	1.153 ^1^	28.83
4.	N17V	KIVKCFVCGK	CF↓VC	0.45	0.284	0.632	15.80
5.	N17A	KIVKCFACGK ^2^	CF↓AC	0.32 ^1^	0.064 ^1^	0.200 ^1^	5.00
6.	N17C	KIVKCFCCGK ^2^	CF↓CC	1.52	0.247	0.162 ^1^	4.05
7.	N17T	KIVKCFTCGK	CF↓TC	0.31	0.017	0.054	1.35
8.	N17Q	KIVKCFQCGK	CF↓QC	0.67	0.035	0.052	1.30
9.	N17S	KIVKCFSCGK ^2^	CF↓SC	0.63	0.004	0.006 ^1^	0.15
10.	N17R	KIVKCFRCGK ^2^	CF↓RC	1.05	0.020	0.019 ^1^	0.48
11.	N17K	KIVKCFKCGK	CF↓KC	0.86 ^1^	0.005 ^1^	0.006 ^1^	0.15
12.	N17E	KIVKCFECGK	CF↓EC	1.21	0.013	0.011	0.28
13.	N17D	KIVKCFDCGK	not hydrolysed				-
14.	N17G	KIVKCFGCGK	CF↓GC	1.34 ^1^	0.002 ^1^	0.001 ^1^	0.03

^1^ These values were published previously [9,25]. ^2^ These mutations were observed in the replication-dependent recovery of the P1′-Gly or P1′-Lys NC-containing mutant viruses [25].

**Table 3:** Proteolytic processing of oligopeptides representing “revertant” mutants of the proximal cleavage site in the HIV-1 NC. The table shows the multiply substituted cleavage site peptides. Modified positions are highlighted with a grey background, the terminal residues added to increase peptide solubility are underlined. The values for wild-type and N17G from Table 2 are also shown here for better comparison, as well.

	Name	Sequence	Cleavage Site	K_m_ (mM)	k_cat_ (s^−1^)	k_cat_/K_m_ ^2^ (mM^−1^ s^−1^)	k_cat_/K_m_ ^2^ Fold-Change	k_cat_/K_m_ ^3^ (mM^−1^ s^−1^)
1.	wild-type	KIVKCFNCGK ^1^	CF↓NC	0.43	0.017	0.040	-	-
14.	N17G	KIVKCFGCGK ^1^	CF↓GC	1.34	0.002	0.001	0.03	-
15.	K14T-N17G	KIVTCFGCGK	n.d. ^4^	-	<0.010	-	-	-
16.	K14I-N17G	RKIVICFGCGKR	IC↓FG	0.035	0.034	0.97 ^5^	24.25	0.51
17.	I14_ins-N17G	KIVKICFGCGKR	IC↓FG	0.070	0.025	0.35	8.75	0.48
18.	N17G-G19I	RKIVKCFGCIKR	n.d. ^4^	-	<0.010	-	-	-
19.	K14I-N17G-G19I	RKIVICFGCIKR	IC↓FG	<0.010	0.015	n.d. ^4^	-	0.48
20.	K14L-N17G	RKIVLCFGCGKR	VL↓CF/LC↓FG	-	n.d. ^4^	-	-	-

^1^ These values were published previously [9]. ^2^ Specificity constant calculated from the K_m_ and k_cat_ values. The fold-changes were determined by comparing the k_cat_/K_m_ values of the mutants to that of the wild-type. ^3^ Specificity constant determined using a competition assay with RPGNF↓LQSRP Gag cleavage site peptide. ^4^ n.d., not determined. ^5^ Increase of substrate concentration above the apparent K_m_ value resulted in a decrease in the measured activity for the substrates.

## Data Availability

The data presented in this study are available on request from the corresponding author.
